# New Insights into the Synergistic Interaction Between Pseudomonas *qingdaonensis* NZ 1 and Silicon to Mitigate Drought Stress in Rice

**DOI:** 10.3390/microorganisms13051046

**Published:** 2025-04-30

**Authors:** Nazree Zainurin, Muhammad Imran, Shifa Shaffique, Muhammad Aaqil Khan, Sang-Mo Kang, Md. Injamum-UL-Hoque, Ashim Kumar Das, Byung-Wook Yun, In-Jung Lee

**Affiliations:** 1 Department of Applied Biosciences, Kyungpook National University, Daegu 41566, Republic of Korea; nazreezainurin@hotmail.com (N.Z.);; 2 Biosafety Division, National Institute of Agriculture Science, Rural Development Administration, Jeonju 54874, Republic of Korea; 3 Department of Chemical and Life Sciences, Qurtuba University, Peshawar 25000, Pakistan

**Keywords:** PGPR, plant–microbe interaction, reactive oxygen species, phytohormones, silicon, synergism, drought stress

## Abstract

The current study assessed the synergistic effects of PGPR and Si in alleviating drought stress in rice. Bacteria were isolated from *Phragmites australis* inhabiting an urban riverbank. Among the isolated strains, *Pseudomonas qingdaonensis* NZ 1 showed promising results under in vitro drought stress induced by PEG-6000 (−0.28 MPa). To further investigate the synergistic effect of *Pseudomonas qingdaonensis* NZ 1 and silicon, a plant growth experiment was conducted comprising the control (dH_2_O) and plants treated with NZ 1, Si (1 mM), and NZ 1+Si under normal and drought stress conditions. The results revealed that NZ 1+Si-treated plants showed improved plant growth parameters, chlorophyll contents, relative water contents, antioxidant potential, and nutrient uptake under normal and drought conditions. Moreover, endogenous abscisic acid and jasmonic acid levels were substantially reduced, while the salicylic acid level was increased in NZ 1+Si-treated plants. Moreover, the relative expression of the ABA metabolic genes *OsNCED3* and *OsCYP707A6* and transcription factors *OsbZIP23* and *OsDREB1B* were significantly altered. Furthermore, the leaf Si, calcium, potassium, and phosphorus contents were increased in NZ 1+Si-treated drought-stressed plants, along with the upregulation of *OsLSi1*. The combined application of NZ 1 and Si offers a sustainable agricultural strategy to effectively mitigate the detrimental effects of drought.

## 1. Introduction

Crops in arid and semi-arid regions are continuously exposed to various abiotic stresses such as drought, salinity, extreme temperatures, and metal toxicity. Among abiotic stresses, drought stress is a major problem that results from long-term, below-average precipitation in a given locality, and it can occur in virtually any climatic regime, in both high and low rainfall areas [1]. Over the past decades, drought has severely impacted crop production, with a recorded loss of ~USD 30 billion worldwide [2]. This is partially due to ROS overproduction; above a certain threshold, damage will occur, which, if the levels remain high, will eventually lead to plant death [3]. To cope with an inadequate water supply, plants alter their growth patterns, structural dynamics, osmotic and hormonal regulation, and delay senescence [4].

The levels of the phytohormone ABA have been found to change in response to drought conditions, increasing the plant’s chance of survival through stomatal regulation [5]. ABA is synthesized by 9-cis-epoxycarotenoid dioxygenase 3 (*OsNCED3*) and catabolized by cytochrome P450 (*OsCYP707A6*). Two main regulatory pathways affecting the gene expression patterns in response to drought have been identified: the ABA-dependent and ABA-independent pathways. The transcription factor (TF) basic region/leucine zipper (bZIP) family is one example of an ABA-independent TF family [6], while dehydration-responsive element binding protein (*DREB*) TFs govern the ABA-independent pathway [7].

Various traditional strategies, such as developing tolerant varieties, crop rotation, and intercropping, have been employed to improve drought resistance. However, these methods are often time-consuming and laborious, and there is a risk of cross-contamination or loss of beneficial host–plant characteristics [8,9]. In contrast, plant growth-promoting rhizobacteria (PGPR) inoculation has emerged as an essential tool in sustainable agriculture, providing a time-efficient and eco-friendly approach to mitigate the effects of drought on plants [8,10]. PGPR inoculation presents an economical and sustainable solution that can enhance crop production during water-deficit periods [9]. The term “plant growth-promoting rhizobacteria” refers to rhizosphere-colonizing bacteria with the ability to stimulate plant growth [11]. They are mainly found in the rhizosphere, where the exudation of photosynthates by roots via rhizodeposition provides an environment rich in nutrients and various organic compounds [12]. Microbial communities thrive in the rhizosphere by feeding on these organic compounds, which include acetic acid, succinic acid, citric acid, lactic acid, malic acid, and propionic acid [13]. By relying on root exudates to fuel their metabolic activity, PGPR and their interaction with plants have been found to alter the plant’s root system architecture [14], contributing to an improved water uptake capability [15], which, in turn, allows for an optimal transpiration rate [16]. Given the persistent issue of freshwater scarcity and the projected increase in the food product demand-to-availability ratio [2], it is imperative to explore alternative agricultural practices. This led to an interest in the utilization of PGPR as an eco-friendly strategy. While it has been reported to generally improve plant growth, several reports have shown that the inoculation of rice with PGPR effectively improves tolerance to various stressors, including water scarcity [17], high salinity [18], heavy metal contamination [19], and extreme temperatures [20]. In addition, Si application has been found to promote growth in rice plants by increasing root growth, thereby improving water uptake and enhancing ion regulation, resulting in upregulated polyamines and increased nutrient intake [21]. Not only can it promote plant growth under normal conditions, but Si has also been found to ameliorate negative stress-related effects in rice grown under adverse conditions, such as water scarcity [22] and high saline conditions [21].

Silicon (Si) is the second most abundant element on Earth, and the application of Si fertilizer has been found to promote plant growth, yield, and quality. Additionally, its non-toxic and pollution-free properties make it an eco-friendly and sustainable agricultural approach [23]. The uptake of Si in plants varies, but rice, being a Si hyperaccumulator, absorbs Si from soils more than any other crop due to its abundance of carrier proteins [24,25]. When applied to rice and sorghum, Si fertilizer was found to increase the leaf transpiration rate during water-scarce conditions [26,27], contributing to an increase in the leaf water content via increased uptake [28,29]. Apart from that, Si improves the structural support of the plant in stress conditions, regulates the secondary metabolites, and facilitates the microbial community to grow [30]. Interestingly, it enhances chlorophyll concentrations [31] and cell wall stability [32] and stimulates the activities of antioxidants [33]. The Si transporter gene *OsLSi* is crucial for transporting Si into the plant root cell, and its deletion in rice plants significantly reduced Si contents [34]. In addition to promoting plant growth, *OsLSi* upregulation has been found to increase rice plant resistance to heavy metal toxicity [35].

Rice is a staple food for a large portion of the world’s population, and its production accounts for the highest proportion of the agricultural industry [36]. Based on a report by FAO via its Rice Market Monitor program, the worldwide yield of rice paddies in 2017–2018 was around 502.9 million tons [37]. As a result of urbanization, there is a shortage of fresh water, and in Asia, rice cultivation requires around 80% of the total freshwater diverted for irrigation [38]. The traditional flooding practices used for rice cultivation have become more challenging, as freshwater availability has to keep up with the demand of population growth despite the impediments of climate change and water pollution [2,39]. Contrasting with other cereal crops, rice is believed to be very susceptible to drought conditions, which alter rice leaf anatomy and ultrastructure, reduce leaf area, and cause leaf rolling, leaf wilting, early senescence, and stomatal closure [40,41].

Drought poses a great risk to food security. Therefore, cultivation techniques and methods must be explored to enhance water use efficiency. Both PGPR inoculation and Si application have independently demonstrated significant potential in alleviating drought’s effects on crops, and both are environmentally friendly. The synergetic effect on experimental plant rice has a tremendous research gap. To the best of our knowledge, this is the first study that reports the synergetic effect of *Pseudomonas qingdaonensis* and Si in an experimental plant rice group japonica cultivar Hwayoung. Therefore, it is important to achieve sustainable agricultural practices while mitigating drought conditions during rice cultivation. This study aims to determine whether the co-application of PGPR (*Pseudomonas qingdaonensis* NZ 1) and Si enhances drought tolerance in rice by modulating antioxidant responses, phytohormone regulation, and nutrient uptake.

## 2. Materials and Methods

### 2.1. Collection, Isolation, and Identification of PGPR Isolates

Bacterial strains were isolated from the rhizospheric soil of *Phragmites australis* inhabiting the riverbank of Geumho River in Daegu, Republic of Korea (35°54′04″ N 128°37′35″ E). All microbes were screened for their plant growth-promoting (PGP) traits, such as phosphate solubilization, the production of exopolysaccharides (EPSs), indole acetic acid (IAA), and siderophore, by following the procedure described by the [18] with slight modifications. Briefly, for the EPS assay, 0.2 g/L Congo red dye was mixed with 50 g/L sucrose, 10 g/L yeast extract, 5 g/L peptone, and 15 g/L agar. After mixing with a magnetic stirrer, the solution was autoclaved at 121 °C for 80 min. Following this, the solution was poured into a Petri dish, and the bacterial strains (1.5 × 10^8^ CFU/mL) were allowed to grow in it. After five days, the growth of microbes against the black background was measured for the presence of EPSs [42].

To determine the presence of IAA, Salkowski’s reagent (1 mL of 0.5 M ferric chloride (FeCl_3_)) and 49 mL of 35% perchloric acid (HCLO_4_) were prepared. A three-day-old culture of the bacterial broth was centrifuged at 10,000 rpm for 10 min, and 1 mL of the supernatant (cell-free culture) and 1 mL of Salkowski’s reagent were mixed. The mixture was placed in a dark room for 30 min. The change in color from transparent to pink was noted [43]. For quantitative measurement of the IAA, 10 mg of pure IAA was mixed with 10 mL of distilled ethanol to prepare 1 mg/mL of stock standard solution, placed at 4 °C, and covered with aluminum foil. Several different serial dilutions of IAA were prepared with distilled water (0, 10, 20, 40, 60, 80, 100 µg/mL). A total of 1 mL of each serial dilution was mixed with 2 mL of Salkowski’s reagent and incubated in the dark at 27 °C. A spectrophotometer was used to measure the optical density at 530 nm. The standard curve was plotted against known IAA concentration and absorbance values. A total of 1 mL of bacterial supernatant was mixed with 1 mL of Salkowski’s reagent, and absorbance was noted at 530 nm, as described by [44,45]. The comparison was made between the standard curve and the bacterial sample. The following equation was used to quantify the IAA in the sample:

Y = mx + c

where Y represents the absorbance, and x shows the IAA concentration.

For the determination of siderophore, the chrome azurol S (CAS) was prepared by mixing three solutions: 60.5 mg CAS was added to 50 mL of distilled water, 1 mM iron solution (FeCl_3_) was prepared with 10 mM HCl, and 72.9 mg hexadecyltrimethylammonium bromide (HDTMA) was mixed in 40 mL of distilled water. After preparing the CAS solution, it was mixed with minimal salt agar base that contains sucrose, magnesium sulfate (MgSO_4_), ammonium sulfate (NH_4_SO_4_), and dipotassium hydrogen phosphate (K_2_HPO_4_). The entire solution was autoclaved after adjusting the pH to 6.8. Following this, assay plates were prepared, and bacterial inoculum 1.5 × 108 CFU/mL was allowed to grow there. The halo zone around the microbial colonies was noted [46].

Five out of thirty-five isolates showed promising results based on these PGP traits and were thus selected to be further analyzed for their potential to produce secondary metabolites and resistance to drought stress. Our main goal is to isolate drought-tolerant microbes.

### 2.2. Preliminary Screening of the Microbial Isolates

Soil and root samples were collected and plated on LB media, yielding 35 distinct bacterial strains. These isolates were initially named according to their source: RSS (rhizospheric soil), CRR (crushed root), and CR (root). The strains were evaluated based on their siderophore, catalase, and EPS production and their phosphate solubilization activities, all of which are known plant growth-promoting traits. Siderophore production by the isolates can be evaluated by the presence of halo zones surrounding the bacterial colonies, with RSS3, RSS19, RSS22, CRR25, CRR37, CRR28, CRR47, CR4, CR24, and CR 28 demonstrating maximum production (Supplemental Figure S1A). Catalase production is measured based on the decolorization of the orange media, with RSS3, RSS19, RSS22, CR4, and CR24 exhibiting the highest levels (Supplemental Figure S1B). Exopolysaccharide production was gauged through colony blackening, with isolates RSS3, CRR25, CR4, CR24, CR36, and CR39 showing maximum production (Supplemental Figure S1C). Phosphate-solubilizing activity was determined based on media transparency, with RSS3, RSS16, CRR35, CRR37, CRR38, CR27, and CR41 displaying the most significant activities (Supplemental Figure S1D). Based on these results, isolates RSS3, RSS22, CRR25, and CR39 consistently demonstrated superior performance across all four parameters and, hence, were examined further in this study. The isolates were subsequently renamed as NZ 1 (RSS3), NZ 2 (CR29), NZ 3 (RSS22), MIJ 1 (CRR25), and MIJ 2 (CRR38) for the remainder of the investigation. The screening test results of the five selected isolates, which are the primary focus of this study, are displayed in Supplemental Figure S2.

### 2.3. Quantification of Secondary Metabolites

The selected five isolates were further examined for their production of phytohormones such as indole-3-acetic acid (IAA) and SA using gas chromatography/mass spectrometry (GC/MS) and HPLC using an octadecylsilyl group column (Hypersil, Thermo Scientific, Waltham, MA, USA), respectively, based on the protocols reported in [47,48]. The in vitro secondary metabolites, such as organic acid (acetic acid, succinic acid, citric acid, lactic acid, malic acid, and propionic acid), and the free amino acid production of the isolates were assessed following the methods detailed in [49,50,51].

### 2.4. Drought Stress Tolerance

Based on the PGP traits, the selected isolates were then assessed via drought tolerance assay using the different concentrations of polyethylene glycol-6000 (PEG-6000), following the steps outlined in [52]. The freshly prepared isolates were grown at 30 °C in a rotating incubator in LB media supplemented with different concentrations (5, 10, 15, and 20%) of PEG-6000 at room temperature, 27 °C. Bacterial cell growth was measured by monitoring their optical density at 600 nm (OD_600_) using a Multiskan GO microplate spectrophotometer (Multiskan Go, Thermo-Fisher Scientific Co., Vantaa, Finland), with 8 h intervals for a total duration of 36 h. Based on their survival trend, isolate NZ 1 was selected for further experimentation.

### 2.5. Molecular Identification

The selected microbial strain was molecularly identified based on the 16S rRNA gene, adopting the methods described by [53] for DNA isolation. The specific primers used for amplification were 24F (5′-AGAGTTTGATC (AC) TGGCTCAG-3′) and 1429R (5′-CGGCTTACCTTGTTACGACTT-3′). For amplification, the denaturation of DNA was carried out at 94 °C for 3 min, followed by the annealing at 50–55 °C for 30 s. After this, the extension was computed at 72 °C for ten minutes. Twenty-five cycles of polymerase chain reaction (PCR) were used [54,55]. The obtained nucleotide sequence of NZ 1 was queried based on sequence similarity against the National Center of Biotechnology Information (NCBI) GenBank database (https://www.ncbi.nlm.nih.gov/, accessed on 12 June 2023) using the Basic Local Alignment Search Tool (BLAST) to identify closely related organisms. A phylogenetic analysis was performed, and a phylogenetic tree was constructed using the neighbor-joining method in MEGA 11: Molecular Evolutionary Genetics Analysis v11 [56].

### 2.6. Screening for the Optimum Si Concentration and Isolate Growth Conditions

To determine the optimal Si concentrations to be used in the pot experiment, the growth performance of 14-day-old rice plants was first evaluated with Si (sodium metasilicate pentahydrate, Na_2_SiO_3_·5H_2_O) additions at five concentrations: (i) 0 mM (control), (ii) 0.5 mM, (iii) 1 mM, (iv) 1.5 mM, and (v) 2 mM. The 1 mM concentration was subsequently selected as this concentration showed the most encouraging growth, with plants in the 1.5 mM and 2 mM treatments showing no significant difference in growth compared to those in the 1 mM Si treatment. Silicon was added via the soil drenching method. Additionally, −0.28 MPa stress by PEG-6000 was used in this screening experiment, as this concentration was found to support the optimal growth of isolate NZ 1.

### 2.7. Pot Experiment

Hwayoung (Oryza sativa japonica) is a widely used wild-type rice cultivar in stress physiology studies, particularly as a control in drought stress experiments involving transgenic and non-transgenic rice lines. It has been previously utilized to evaluate the metabolic and physiological responses of rice to water-deficit conditions [57,58]. While Hwayoung is not explicitly classified as a drought-tolerant variety, its well-documented baseline response to drought stress makes it a suitable reference for assessing the effects of plant growth-promoting rhizobacteria (PGPR) and silicon under drought conditions. Using Hwayoung as the control allows for meaningful comparisons and aligns our study with existing research methodologies in drought stress physiology [59].

Hwabyeongbyo rice seeds cultivar was provided by the Crop Physiology Laboratory, Kyungpook National University. The obtained seeds were initially surface sterilized in 0.05% antifungal agent (Prochloraz 25%, Samgong Co. Ltd., Daegue, Republic of Korea) and rinsed and soaked in dH_2_O for 48 h, with the water being replaced after 24 h. The germinated seeds were transferred to 190-cell horticultural planters filled with autoclaved Daepoong20 nursery soil (Samhwa Green Tech Ltd., Ulsan, Republic of Korea), which was composed of 50% red clay/loess, 30% vermiculite, 19.5% biotite, 0.5% fertilizer, and other materials. The seedlings were grown in a growth chamber at 65–75% relative humidity with a 16 h light period (200 umol photons m^−2^ s^−1^) at 26 °C and an 8 h dark period at 24 °C. Two weeks after germination, seedlings of roughly uniform size were transplanted to 12 × 10 cm pots containing approximately 400 g of Daepoong20 soil.

The plants were divided into two groups: a non-stressed group—with treatments consisting of (i) dH_2_O only (control), (ii) isolate NZ 1, (iii) 1 mM of Si, and (iv) NZ 1 + 1 mM Si—and a drought-stressed group—with treatments consisting of (i) −0.28 MPa (drought-stressed control), −0.28 MPa + NZ 1, (iii) −0.28 MPa + 1 mM Si, and (iv) −0.28 MPa + NZ 1 + 1 mM Si. All treatments were administered via the soil drenching method, and the drought conditions were imposed for seven days. PEG 6000 was used to induce the artificial stress in experimental plants; 1 × 10^8^ CFU/mL microbial pellet was used to induce the plant–microbial interaction. Before harvesting, the morphological and physiological parameters of each plant were recorded and stored at −80 °C for further assessment.

### 2.8. Assessment of Plant Morphological Parameters and Non-Invasive Physiological Responses

Prior to harvesting, morphological growth parameters and the (RWC) percentages of fresh leaf samples were determined following the method previously described by [60]. RWC was calculated using the following formula:

$$\mathrm{RWC}=\frac{(\mathrm{fresh}\;\mathrm{weight}-\mathrm{dry}\;\mathrm{weight})}{\left(\mathrm{turgid}\;\mathrm{weight}-\mathrm{dry}\;\mathrm{weight}\right)}\times 100\%$$

Chlorophyll contents were measured with a CCM-300 chlorophyll content meter (ADC BioScientific Ltd., Hoddesdon, UK). Leaf hyperspectral reflectance was determined using a PolyPen RP 410 (Photon Systems Instruments, Drásov, Czech Republic), and selected indices were calculated based on the hyperspectral data (Table 1). Measurements were taken from the tip of the longest leaf of each plant within the same hour.

### 2.9. Determination of Hydrogen Peroxide, Superoxide Anion, and Malondialdehyde Production

Hydrogen peroxide (H_2_O_2_) content was analyzed using the methods described by [63]. In brief, freeze-dried leaf samples were mixed with 0.1% trichloroacetic acid (TCA) and homogenized, and the resultant suspension was mixed with a 1 M potassium iodide (KI) and 10 mM sodium phosphate buffer (pH 7.0) solution. The absorbance was then measured at 390 nm, and the H_2_O_2_ content was calculated using the extinction coefficient (ε) 0.28 mM cm^−1^ and expressed as µmol g^−1^ DW. Superoxide anion (O_2_^•−^) production was determined using the method described by [64]. Malondialdehyde (MDA) quantification was conducted according to the method described by [65]. The absorbance of the MDA assay supernatant was read at two wavelengths: 532 and 600 nm, and MDA content was calculated using the formula *R*_1_ − *R*_2_ and expressed as µmol g^−1^ DW. Reading 1 was at 532 nm, and Reading 2 was at 600 nm.

MDA content = *R*_1_ − *R*_2_/Extinction coefficient × Path length

### 2.10. Quantification of the Plants’ Antioxidant Enzyme Activities

To further examine the production of antioxidants enzymes, the production of superoxide dismutase (SOD), catalase (CAT), glutathione GSH, and ascorbate peroxidase (APX) in the experimental plants was quantified. These analyses were carried out in plate-based assays, in 96-well plates, using a Multiskan Go microplate spectrophotometer (Multiskan Go, Thermo-Fisher Scientific Co., Vantaa, Finland) to measure absorption at the appropriate wavelength for the respective assay.

To quantify the SOD activities of the plants from each treatment, the methods of [66,67] were followed. Briefly, freeze-dried leaf samples were extracted using SOD extraction buffer, and the supernatant was used to obtain absorbance readings at 420 nm from three solutions: 50 µL supernatant + 150 µL extraction buffer + 50 µL pyrogallol (*A*), 50 µL supernatant + 200 µL extraction buffer (*B*), and 150 µL extraction buffer + 50 µL pyrogallol (*C*). Using these readings, the SOD units were determined according to the following equation:

$$\mathrm{SOD}\;\mathrm{units}=\left(1-\frac{\left(A-B\right)}{C}\right)\times 100$$

To quantify CAT activity, we used the methods described by [68,69]. Briefly, freeze-dried leaf samples were homogenized in CAT extraction solution, the resulting suspension was mixed with H_2_O_2_ and phosphate buffer (pH 7.0), and a supernatant was obtained. Then, an enzyme extract was added to the reaction mixture to initiate the reaction. The resulting absorbance was measured at 240 nm, and CAT activity was estimated using a standard curve. To quantify GSH activity, the method of [70] was employed, while APX activities were measured using the method outlined in [71].

### 2.11. Quantification of the Plants’ Soluble Sugars and Amino Acids Content

Soluble sugars were extracted from the freeze-dried samples in accordance with the protocols of [72]. Samples were extracted, and the supernatants were analyzed with an Alltech 3300 ESLD detector (Alltech, Deerfield, IL, USA) attached to an HPLC system (Waters Corp., Milford, MA, USA). The methodologies described in [50] were adopted in quantifying the amino acid content of the plant samples. In brief, freeze-dried samples were digested with 6N HCl under vacuum conditions before being homogenized in 0.02 N HCl and filtered through a 0.22 µM Millipore filter. An atomic Amino Acid Analyzer L-8900 (Hitachi, Japan) was used for the analysis, and the concentrations were determined by comparing the observed retention times and peaks to those of established standards.

### 2.12. Quantification of the Plants’ Nutrient Levels

Nutrient level quantification was carried out using the methods described by [73]. Briefly, freeze-dried leaf samples were soaked in 0.5 M HCl and heated for 8 h at 100 °C. The obtained digested samples were subjected to an ICP-MS (Optima 7900DV; Perkin-Elmer, Waltham, MA, USA) analysis to assess the levels of essential ions generated under the different treatments.

### 2.13. Quantification of the Plants’ Endogenous Phytohormones ABA, JA, and SA

Endogenous ABA was extracted and quantified following the method previously described by [74], with slight modifications described by [53]. The fractions were methylated before quantification through GC–MS (6890N network gas chromatography system, Agilent Technologies, Hong Kong), using Me-[^2^H_6_]-ABA as an internal ABA standard. The signal ions (*m*/*z* 162 and 190 for Me-ABA and *m*/*z* 166 and 194 for Me-[^2^H_6_]-ABA) were monitored using software from ThermoQuest Corp. (Manchester, UK, D.03.00.552). The extraction and quantification of jasmonic acid (JA) were conducted in accordance with the method of [75], with slight modifications. An internal JA standard ([9,10-^2^H_2_]-9,10-dihydro-JA) was used, and the fractions were esterified with diazomethane, methylated, and analyzed using GC-MS (6890N network GC system). The ion fragments were monitored at m/z 83 AMU corresponding to the base peaks of JA and [9,10-^2^H_2_]-9,10-dihydro-JA. The endogenous JA contents were estimated by comparing the peak areas of the sample with those of their respective standards. The extraction and quantification of SA were performed using methods adopted from [47]. The samples were filtered through a 0.22 µ Millipore filter (DISMIC—25CS, Advante, Kyoto, Japan) before injection into a high-performance Hypersil octadecylsilyl group C18 column for reverse-phase HPLC (Thermo Scientific). The detection of SA was performed using a spectrofluorometer (RF-10 AXL, Shimadzu, Kyoto, Japan), and the SA concentration in each sample was determined based on the peak values of known standards.

### 2.14. Relative Gene Expression

The relative expression of several genes, such as *OsNCED3*, *OsCYP707A6*, *OsbZIP23*, *OsDREB1B*, and *OsLSi1* of the plant samples, was determined using real-time quantitative reverse transcription PCR (qRT-PCR). The extraction of RNA from the frozen leaf samples was carried out using TRIzol, and cDNA synthesis was conducted with a DiaStar^TM^ RT kit (SolGent, Daejeon, Republic of Korea). The qRT-PCR was performed in an ECO^TM^ real-time PCR system using reaction mixtures including 2X real-time PCR Master Mix (including SYBR^®^Green I BioFACT^TM^, Daejeon, Korea), the previously synthesized cDNA as the template, and gene-specific forward and reverse primers. After an initial denaturing step at 95 °C for 5 min, the samples were subjected to 40 cycles of denaturing at 94 °C for 20s, annealing at 58.5 °C for 40, and extension at 72 °C for 1 min, followed by a final extension step at 72 °C for 5 min. The *OsActin* gene was used as the housekeeping gene. The genes and their corresponding primers are detailed in Supplemental Table S1.

### 2.15. Statistical Analysis

The data analysis was conducted using RStudio v2023.12.1+402, running R v4.2.2 [76]. One-way and two-way analyses of variance (ANOVAs) were performed to determine the significance of main effects, with Tukey’s post hoc tests used to compare means. A *p*-value < 0.05 was considered statistically significant for all tests. Graphic visualizations were produced using the ggplot2 package (version3.5.1). Mega X (version 11.0.13) was used to create the phylogenetic tree.

## 3. Results

### 3.1. In Vitro Microbial Antioxidant, Phytohormone, Organic Acid, and Amino Acids Production and Drought Stress Tolerance

**Figure 1:**
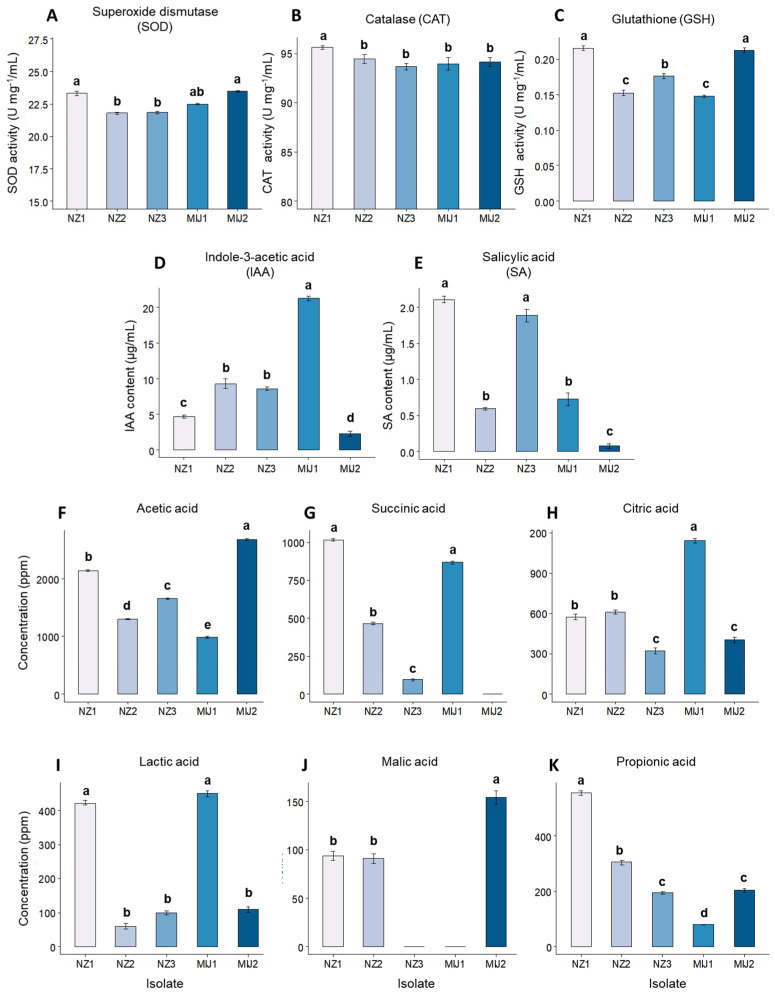
The isolates’ production of the antioxidants (**A**) superoxide dismutase, (**B**) catalase, and (**C**) glutathione; the phytohormones (**D**) indole-3- acetic acid and (**E**) salicylic acid; and the organic acids (**F**) acetic acid, (**G**) succinic acid, (**H**) citric acid, (**I**) lactic acid, (**J**) malic acid, and (**K**) propionic acid. Each bar represents a mean, and error bars represent the standard error of the mean based on 5 replicates. Bars with the same letter are not significantly different from each other, as evaluated using Tukey’s HSD post hoc tests (*p* ≤ 0.05).

The five selected isolates, NZ 1, NZ 2, NZ 3, MIJ 1, and MIJ 2, were assessed for their production of antioxidants and secondary metabolites and drought-stress tolerance. The antioxidant, phytohormone, and organic acid production results are visualized in Figure 1. Antioxidant assays revealed that NZ 1 and MIJ 2 produced the highest levels of SOD, NZ 1 displayed the highest CAT production, and NZ 1, NZ 2, and MIJ 2 showed the highest GSH production. In phytohormone production assays, MIJ 1 produced the highest levels of IAA, while NZ 1 and NZ 3 exhibited the highest SA production. The ability to produce organic acids varied greatly among the isolates. Isolate MIJ 1 produced the highest amounts of citric and lactic acids, MIJ 2 produced the highest amounts of malic and acetic acids, and NZ 1 produced the highest amounts of succinic and propionic acids (Figure 1). Notably, NZ 3 and MIJ 2 did not produce any malic acid, and no succinic acid was produced by MIJ 1.

The free amino acid analysis showed that isolate NZ 1 produced the highest amounts of most amino acids, including aspartic acid, serine, glycine, valine, methionine, isoleucine, tyrosine, phenylalanine, and arginine, as shown in Table 2. On the other hand, isolate NZ 3 led in lysine production, while MIJ 2 produced the most glutamic acid, cysteine, lysine, and proline. Overall, NZ 1 produced the highest amounts of amino acids.

To assess their drought tolerance, the five strains were tested for their capability to grow in five different concentrations of PEG-6000: (−0.05, −0.14, −0.28, −0.5) MPa at room temperature (27c). The experiment was conducted for 32 h, with OD_600_ readings taken every 8 h. No differences were observed between strains in the 5 or 10% PEG-6000 treatments, but differences were observed in the −0.28 MPa treatment, where NZ 1 showed the highest OD_600_ values. A graphical representation of the microbial growth results is given in Supplementary Figure S3.

### 3.2. Phylogenetic Identification of Isolate NZ 1

The NZ1 was selected for molecular identification and further experiments based on its performance in producing antioxidants, phytohormones, organic acids, and amino acids. The molecular identification and phylogenetic analysis of NZ 1 were conducted through 16s rRNA amplification and sequencing, and the sequence was compared with known nucleotide sequences in the NCBI GenBank database using BLAST. This search found that NZ 1 showed 99% similarities with existing *Pseudomonas qingdaonensis* sequences. Using the nucleotide sequences of closely related species gathered from the GenBank database, a neighbor-joining phylogenetic tree was constructed, as presented in Supplementary Figure S4. The nucleotide sequence of NZ 1 is available in the NCBI GenBank database under the accession number OR122470.

### 3.3. Plant Morphological Analysis

**Figure 2:**
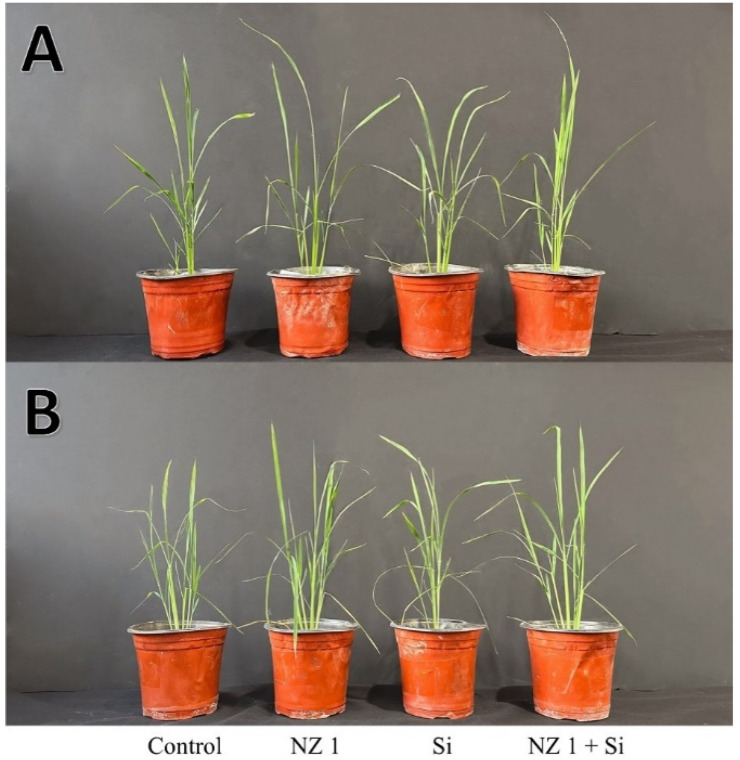
Representative images of rice plants under (**A**) normal and (**B**) drought conditions after recovery from the drought.

The effects of NZ 1 inoculation and Si soil application to rice plants, particularly on their agronomic traits, were evaluated under normal and drought conditions. A Si concentration of 1 mM was chosen for this experiment screening the results of preliminary trials. This study revealed that the co-application of NZ 1 and Si significantly enhanced agronomic traits, especially during drought conditions. The inoculation of isolate NZ 1 alone notably improved several agronomic traits during drought conditions, especially the tiller number, tiller height, LAI, and root and shoot weights, as shown in Figure 2 and Table 3. More specifically, NZ 1 significantly enhanced tiller number and height by approximately 40%, while also improving the other agronomic traits. Similarly, the sole application of Si improved these traits as well, with notable increases in the tiller number and height, leaf area index (LAI), root and shoot weights. However, even greater improvements were seen when NZ 1 and Si were added to the soil simultaneously during drought conditions. The combined application enhanced the tiller number and height, LAI, and seedling characteristics, surpassing the effects of separate applications.

### 3.4. Chlorophyll Content, NDVI, ARI1, and RWC

**Figure 3:**
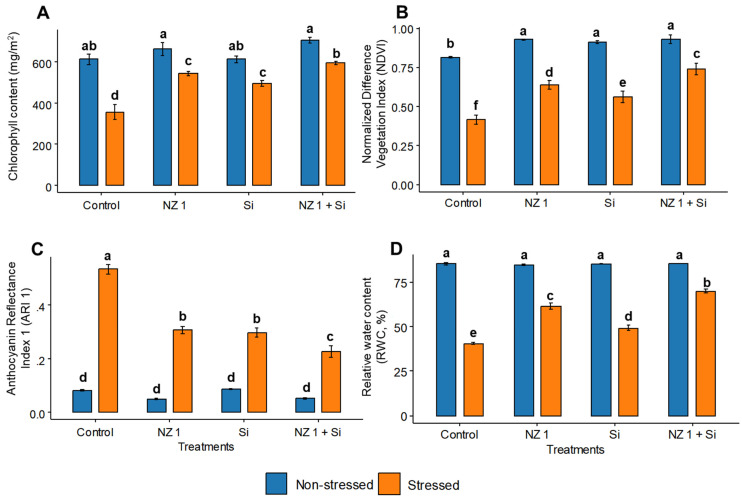
The (**A**) chlorophyll contents and (**B**) normalized difference vegetation index (NDVI), (**C**) anthocyanin reflectance index 1 (ARI 1), and (**D**) percent relative water content (RWC) values of non-stressed and drought-stressed plants under NZ 1 and Si treatments. Each bar represents a mean, and error bars represent the standard error of the mean (*n* = 5). Bars with the same letter are not significantly different, as evaluated using Tukey’s HSD post hoc tests (*p* ≤ 0.05).

Significant improvements are recorded in the plants’ chlorophyll contents and normalized difference vegetation index (NDVI), anthocyanin reflectance index 1 (ARI 1), and percent relative water content (RWC) values when rice plants were treated with NZ 1 and Si simultaneously (Figure 3). Under normal conditions, compared to the control, the chlorophyll content and NDVI were significantly increased, by 15 and 14%, respectively, when treated with both NZ 1 and Si, while the ARI1 and RWC showed a non-significant increment of 5%. Under drought conditions, the chlorophyll content, NDVI, and RWC of the control plants were found to be significantly lower, by 42, 49, and 52%, respectively, while the ARI1 was significantly greater by fivefold. However, the co-application of NZ 1 and Si produced the greatest improvements in these four parameters during drought conditions, with significant increases of 67, 78, and 72% in the chlorophyll content, NDVI, and RWC, respectively, and a noteworthy decrease of 58% in the ARI1, when compared to the drought-stressed control.

### 3.5. ROS and Antioxidants Contents

**Figure 4:**
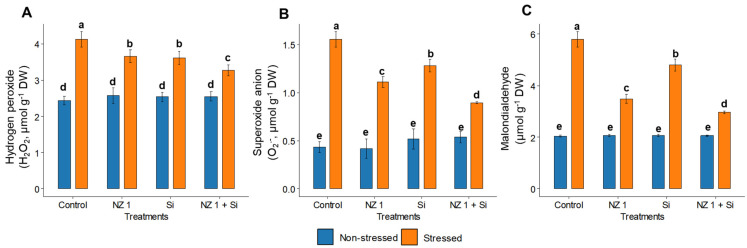
(**A**) Hydrogen peroxide (H_2_O_2_), (**B**) superoxide anion (O_2_^•−^), and (**C**) malondialdehyde (MDA) levels in the leaves of rice plants under NZ 1 and Si treatments and under normal and drought-stress conditions. Each bar represents a mean, and the error bars represent the standard error of the mean. Bars with the same letter are not significantly different from each other, as evaluated using Tukey’s HSD post hoc tests (*p* ≤ 0.05).

**Figure 5:**
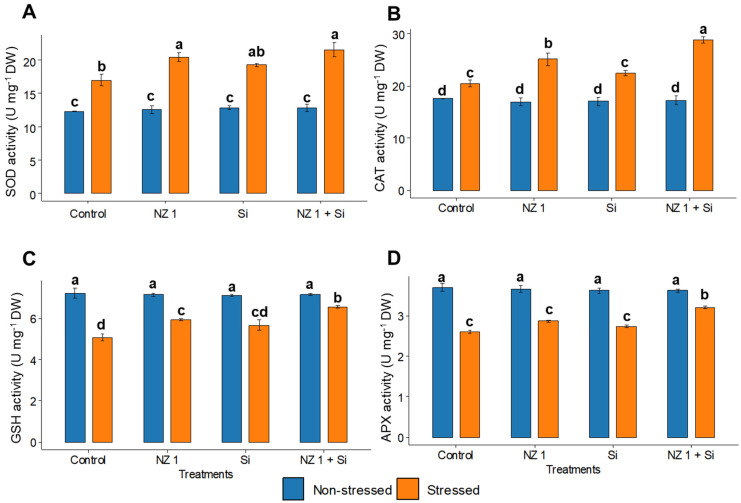
(**A**) Superoxide dismutase (SOD), (**B**) catalase (CAT), (**C**) reduced glutathione (GSH), and (**D**) ascorbate peroxidase (APX) contents in the leaves of rice plants under NZ 1 and Si treatments and under normal and drought-stressed conditions. Each bar represents a mean, and the error bars represent the standard error of the mean (*n* = 5). Bars with the same letter are not significantly different, as evaluated using Tukey’s HSD post hoc tests (*p* ≤ 0.05).

Under drought conditions, the levels of H_2_O_2_, O_2_^•−^, and the oxidative stress biomarker, MDA, in the drought control plants were found to be significantly elevated, increasing by 69, 260, and 183%, respectively, when compared to the non-stressed control rice plants (Figure 4). The separate applications of NZ 1 and Si each modulated the levels of H_2_O_2_, O_2_^•−^, and MDA. However, the most substantial reductions were observed with their simultaneous application, which produced significant reductions of 10%, 43%, and 49%, respectively, compared to the drought-stressed control. Looking at antioxidants, drought conditions were found to significantly increase the SOD and CAT levels of the control plants, by 66% and 16%, respectively, while GSH and APX levels were significantly decreased, by 32% and 30%, respectively (Figure 5). When NZ 1 and Si were simultaneously applied during drought conditions, the levels of these antioxidants were found to be enhanced by 20% in SOD, 42% in CAT, 24% in GSH, and 32% in APX, when compared to those of drought control.

### 3.6. Phytohormones and ABA Metabolic Gene Expression

**Figure 6:**
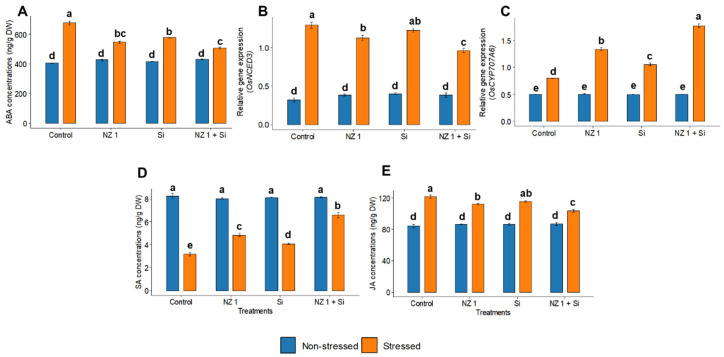
Production of the phytohormone (**A**) ABA, the relative gene expression of (**B**) *OsNCED3* and (**C**) *OsCYP707A6*, and production of the phytohormones (**D**) salicylic acid (SA) and (**E**) jasmonic acid (JA) in the leaves of rice plants under NZ 1 and Si treatments and under normal and drought-stressed conditions. Each bar represents a mean, and the error bars represent the standard error of the mean (*n* = 5). Bars with the same letter are not significantly different, as evaluated using Tukey’s HSD post hoc tests (*p* ≤ 0.05).

Under drought stress, the synthesis of the phytohormone ABA and the relative gene expression of the ABA biosynthesis gene, *OsNCED3*, were found to be increased by 67% and 171%, respectively, in the control plants (Figure 6). However, the combined application of NZ 1 and Si reduced the synthesis of ABA and expression of *OsNCED3* by 25% and 47%, respectively, relative to that of the drought-stressed control. At the same time, the relative gene expression of the ABA catabolic gene *OsCYP707A6* was found to be significantly upregulated, by 121%, when drought-stressed rice plants were treated with NZ 1 and Si simultaneously. Additionally, the synthesis of the phytohormones SA and JA was found to be modulated when rice plants were treated during drought conditions, with a significant increment of 91% and a decrement of 21%, respectively.

### 3.7. Osmolytes Content

**Figure 7:**
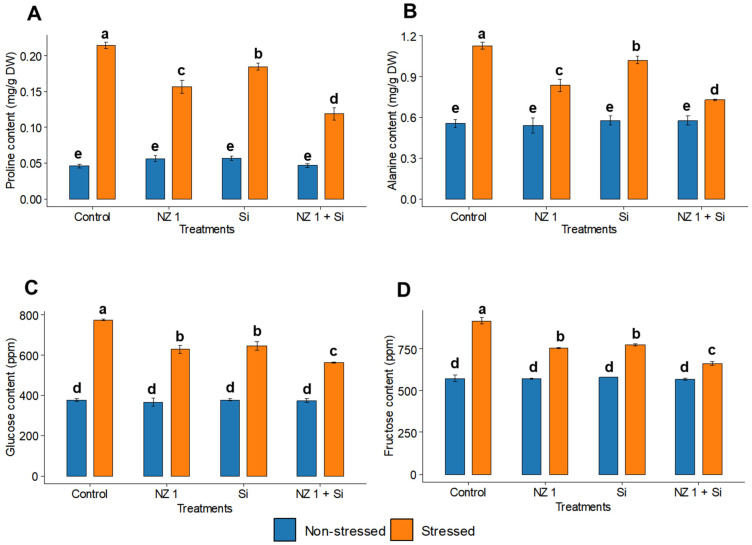
(**A**) Proline, (**B**) alanine, (**C**) glucose, and (**D**) fructose contents in the leaves of rice plants under NZ 1 and Si treatments and under normal and drought-stressed conditions. Each bar represents a mean, and error bars represent the standard error of the mean (*n* = 5). Bars with the same letter are not significantly different, as evaluated using Tukey’s HSD post hoc tests (*p* ≤ 0.05).

Under drought conditions, the levels of the amino acids like proline and alanine exhibited significant increases, by 5-fold and 1-fold, respectively, in the rice plants of the control treatments (Figure 7A,B). However, the co-application of NZ 1 and Si in the drought-stressed group managed to reduce the synthesis of both amino acids by 48% and 30%, respectively. Furthermore, the carbohydrate contents, including glucose and fructose, of the rice plants were also found to be significantly increased under drought conditions, by 105% and 58%, respectively, when comparing the drought-stressed and non-stressed control plants (Figure 7C,D). With the co-application of NZ 1 and Si, the contents of glucose and fructose were found to be significantly reduced, by 24% and 30%, respectively, when compared to the non-treated drought-stressed rice plants.

### 3.8. Potassium, Calcium, Phosphorus, and Si Contents and the Relative Gene Expression of the Si Transporter OsLSi1

**Figure 8:**
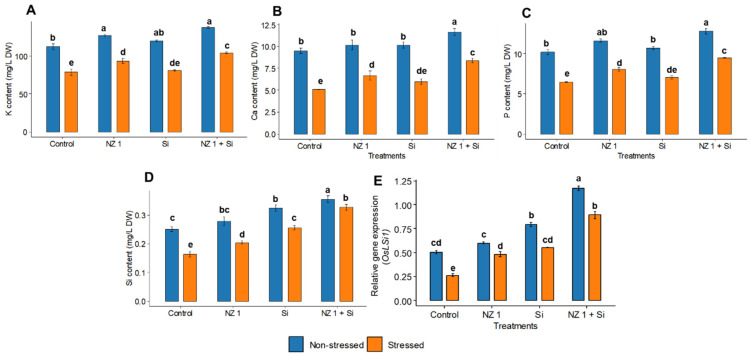
(**A**) Potassium (K), (**B**) calcium (Ca), (**C**) phosphorus (P), and (**D**) silicon (Si) contents, and (**E**) the relative gene expression of *OsLSi1*, in the leaves of rice plants under NZ 1 and silicon (Si) treatments and under normal and drought-stressed conditions. Each bar represents a mean, and error bars represent the standard error of the mean (*n* = 5). Bars with the same letters are not significantly different, as evaluated using Tukey’s HSD post hoc tests (*p* ≤ 0.05).

Separate applications of NZ 1 and Si improved the nutrient contents of rice plants during non-stressed conditions. However, the most significant increments were recorded when NZ 1 and Si were applied simultaneously, which increased potassium (K), calcium (Ca), phosphorus (P), and Si contents by 23, 14, 26, and 42%, respectively (Figure 8A–C). Drought conditions significantly decreased the contents of these elements (K, Ca, P, and Si) by 43, 30, 27, and 35%, respectively. However, under drought stress conditions, the combined treatment of NZ 1 and Si improved the uptake of K, Ca, P, and Si by 54%, 27%, 19%, and 100%, respectively. The relative expression of the Si transporter *OsLSi1* was found to be enhanced when Si was applied simultaneously with NZ 1 (Figure 8D). Under normal conditions, the application of Si coupled with NZ 1 induced a significant upregulation of *OsLSi1* expression, by 132%, as opposed to an upregulation of 57% when Si was applied alone. Similarly, under drought conditions, the upregulation of *OsLSi1* also significantly increased, by 239%, when Si was applied simultaneously with NZ 1; in comparison, the sole application of Si resulted in a lesser, albeit still significant, increase of 100% (Figure 8E).

### 3.9. Expression of Transcription Factors OsbZIP23 and OsDREB1B and Si Transporter OsLSi1

**Figure 9:**
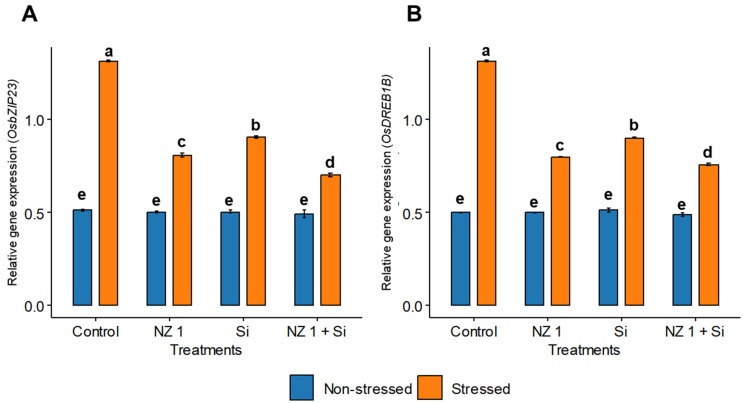
The relative gene expression of (**A**) *OsbZIP23* and (**B**) *OsDREB1B* in the leaves of rice plants under NZ 1 and silicon (Si) treatments and under normal and drought-stressed conditions. Each bar represents a mean, and error bars represent the standard error of the mean. Bars with the same letter are not significantly different, as evaluated using Tukey’s HSD post hoc tests (*p* ≤ 0.05).

The relative gene expressions of the transcription factors, i.e., *OsbZIP23* and *OsDREB1B*, were found to be significantly increased under drought conditions, with increases of 155% and 161%, respectively, when comparing the drought-stressed to the non-stressed control plants (Figure 9). In drought-stressed rice plants, the simultaneous application of NZ 1 and Si significantly downregulated the expression of these genes by 47% and 42%, respectively.

## 4. Discussion

As a semi-aquatic plant, rice demands adequate water for its growth and development. Due to this, it uses up to 43% of the water used for irrigation globally, and more than 50% of the available freshwater is diverted for irrigation in Asia [77]. A lack of water will result in stunted growth and development, eventually leading to lower crop productivity. In this study, rice plant morphological growth, chlorophyll contents, NDVI, and RWC were negatively affected under drought conditions, but a simultaneous application of the PGPR strain NZ 1 and a Si fertilizer alleviated these effects and significantly reduced anthocyanin accumulation. These effects could be attributed to PGPRs’ production of exopolysaccharides, a major component of microbial biofilms, allowing them to maintain rhizosphere water potential and, at the same time, promote plant growth [78,79,80]. This contention is supported by several studies, which have also shown that exopolysaccharide-producing PGPR strains isolated from the rhizospheres of plants inhabiting harsh conditions, such as beaches [81] and deserts [82], improve plant growth under stressed conditions. On the other hand, the application of Si has been found to enhance the chlorophyll content in plants even under stress conditions [83] that could be preventing thylakoid and chloroplast membrane damage, thus protecting tissues and enhancing plant growth under drought conditions [84,85].

Water scarcity influences a plant’s amino acid, carbohydrate, and osmolyte contents and its inorganic nutrient content. In rice plants, it was reported that greater osmolyte accumulation occurs under water-deficit conditions than under normal irrigated conditions [86]. This accumulation could be attributed to their role in protective cellular functions and the maintenance of cellular structures, hence avoiding damage and increasing stress tolerance [87,88]. In this study, osmolyte accumulation was observed in drought-stressed rice plants. However, the simultaneous application of NZ1 and Si significantly reduced drought stress-induced proline, alanine, and soluble sugar accumulation. This may be due to the enhanced expression of the aquaporin gene *OsLSI1*, which could increase or maintain a stressed plant’s root water uptake, resulting from the PGPR and Si application [83]. The increase in proline accumulation is directly related to drought tolerance, as proline helps plants maintain stomatal conductance and stomatal turgidity [89] while also inhibiting programmed cell death due to oxidative stress under adverse conditions [90]. On the other hand, soluble sugar accumulation under drought conditions protects cell membrane integrity and acts as an osmoprotectant [7,91].

Nutrients such as phosphorus are not readily available in the rhizosphere. Sufficient phosphate uptake promotes plant growth through various mechanisms, enhancing root formation and development [92,93]. Solubilized phosphate availability in the soil can be influenced by PGPR due to their phosphate-solubilizing properties and the secretion of organic acids that transform insoluble phosphates into readily available forms [94,95]. Several reports have shown that phosphate-solubilizing [96] and organic acid-producing *Azospirillum* sp. [97] and *Bacillus pumilus SH-9* [81] improve nutrient availability, hence promoting growth in adverse conditions. Si, on the other hand, improves the functionality of membrane transporters, improves electrochemical gradients, and enhances ion channel and carrier activities [98]. One study found that 150 kg/ha of Si improved the growth, yield components, and grain yield of rice [99]. LOW SILICON 1 (*LSi1*), a Si influx transporter, is responsible for the absorption of Si, and the expression of *LSi1* is positively correlated with Si absorption [29,100]. The results of this study support these previous findings, as the upregulation of Si coincided with higher Si content in the plants. Moreover, combining PGPR inoculation (*Bacillus* spp.) and Si application has been found to improve nutrient uptake and, hence, increase plant growth parameters in plants under salt stress, which, along with our findings, provides strong evidence of their beneficial impacts on plant growth during adverse conditions [101].

ROS are produced naturally by plants as by-products of cellular metabolism, but their overproduction under adverse conditions may lead to oxidative stress, causing cellular degradation and toxicity [102]. To avoid oxidative damage, plants produce antioxidants to reduce stress levels by transforming ROS into more stable, non-harmful molecules [103,104]. Under drought conditions, the simultaneous NZ 1 and Si treatment stimulated the production of the antioxidants SOD, CAT, GSH, and APX and reduced levels of H_2_O_2_, O_2_^•−^, and MDA. This may be attributed to NZ 1 increasing the synthesis and accumulation of antioxidants inside the plant, as has been seen with other PGPR species [105,106,107], notably during adverse conditions [108,109]. Si can also eliminate ROS in this case by maintaining carbon fixation and enhancing antioxidant levels in stressed plants [110,111]. Enhanced antioxidant activities following the application of Si have been seen in drought-stressed rice [112], wheat [113], and sugar cane [114].

Under stressed conditions, ROS accumulation triggers the upregulation of ABA, an essential stress hormone that governs the closing of stomata to conserve water, thereby facilitating a plant’s tolerance to unfavorable conditions [5]. The biosynthesis of ABA is facilitated by the catalytic action of 9-cis-epoxycarotenoid dioxygenase (*NCED*) family enzymes [115], and their production could be influenced by decreases in leaf turgor due to limited water availability and the accumulation of ROS [116]. In the present study, ABA production significantly increased during drought conditions, along with the upregulation of *OsNCED3*. Under the same conditions, the expression of *OsDREB1B* and *OsbZIP23* was also significantly upregulated. These genes enhance ABA signals to protect plants during adverse conditions, and their upregulation is heavily involved in the drought tolerance of the plants [117,118,119]. Drought-stressed rice plants treated with both NZ 1 and Si exhibited a significantly lowered level of ABA and the downregulation of the *OsNCED3*, *OsDREB1B*, and *OsbZIP23* genes. At the same time, the expression of the ABA catabolic gene *OsCYP707A6* was upregulated. This gene functions to maintain ABA levels, and its upregulation is triggered when stress is removed, allowing for rapid ABA degradation [120,121,122,123]. These changes could be associated with improved water availability for the drought-stressed plants when their soil was treated with NZ 1 and Si.

The phytohormone JA holds a significant role in plant responses to abiotic stressors and stress tolerance through ROS removal and osmoprotectant production [124]. It is well documented that ABA affects JA synthesis and accumulation, and that these phytohormones work synergistically in regulating rice growth and the development of abiotic stress responses [123,125,126,127]. Salicylic acid is an essential endogenous phytohormone, and together with ABA and JA, it is responsible for regulating protein expression and plant defense systems. However, SA and JA have an antagonistic relationship during stress conditions, and this study agrees with previous studies demonstrating an SA–JA antagonistic crosstalk [128,129]. Several reports have shown that exogenous applications of SA can contribute to plant growth enhancement through the upregulation of photosynthesis and antioxidant activity during drought conditions [130,131,132]. Since NZ 1 was found to produce a moderate amount of SA and IAA, its inoculation could alleviate drought stress in plants, as has been reported in several studies using SA-producing PGPR [51,133,134] and IAA-producing PGPR [135,136] as inoculants, which have demonstrated improved morphological growth. Si applications can also regulate the biosynthesis of endogenous ABA, SA, and JA, which actively promote plant growth and development during stress conditions [137,138]. The present study is different from previous studies because it is the first study that reports the drought tolerance in *Pseudomonas qingdaonensis* species.

## 5. Conclusions and Future Aspects

Climate change is an issue not only for crops but also for human beings. If crop productivity plummets due to increasingly unfavorable conditions, such as drought, we may face a dwindling food supply in the future. With the projected rise of the human population and lower freshwater availability, alternative outlooks and new eco-friendly agriculture practices must be explored. This study explored the use of a novel PGPR, *Pseudomonas qingdaonensis* NZ 1, combined with Si fertilization, to ameliorate the effects of drought conditions in rice and produced encouraging results. The sole application of either isolate NZ 1 or Si enhanced the growth of rice under drought conditions. Still, the most significant positive results were seen when NZ 1 and Si were applied together. Their co-application to soil under drought conditions resulted in improved agricultural production, vegetative indices, and nutrient contents in the rice plants. This could stem from the modulation of antioxidants, ROS, and osmolyte production. Furthermore, modulation in phytohormone ABA expression pathways was affected by the combined NZ 1 and Si application, and these changes promoted rice plant growth under drought conditions. The co-application of NZ 1 and Si has been promised as a potential treatment for enhancing plant production even during drought. These two elements can be used in the production of biostimulants, providing a more sustainable, eco-friendly approach to agriculture, and may be especially useful for managing adverse conditions.

## Figures and Tables

**Table 1:** Reflectance indices calculated by the PolyPen RP 410 are based on hyperspectral reflectance. NIR represents light reflected in the near-infrared spectrum, while RED shows light reflected in the red range of the spectrum.

Reflectance Index	Equation	Reference
NDVI (normalized difference vegetation index)	$\mathrm{NDVI}=\frac{{(R}_{\mathrm{NIR}}-R_{\mathrm{RED}})}{R_{\mathrm{NIR}}+R_{\mathrm{RED}}}\;$	[61]
ARI1 (anthocyanin reflectance index 1)	$\mathrm{ARI}1=\frac{1}{R_{550}}-\frac{1}{R_{700}}$	[62]

**Table 2:** Free amino acid production by the five selected isolates.

Amino Acid Content (mg/g) ^†^	Isolate				
	NZ 1	NZ 2	NZ 3	MIJ 1	MIJ 2
Asp	7.54 ± 0.03 ^a^	6.23 ± 0.10 ^c^	7.38 ± 0.12 ^a^	7.12 ± 0.58 ^b^	7.10 ± 0.29 ^b^
Thr	2.42 ± 0.02 ^b^	3.06 ± 0.05 ^a^	2.24 ± 0.04 ^c^	2.45 ± 0.05 ^b^	2.20 ± 0.20 ^c^
Ser	2.25 ± 0.05 ^b^	2.05 ± 0.03 ^c^	1.91 ± 0.03 ^c^	2.18 ± 0.11 ^b^	4.57 ± 1.76 ^a^
Glu	22.97 ± 0.37 ^a^	20.54 ± 0.33 ^b^	23.03 ± 0.37 ^a^	22.07 ± 1.00 ^a^	14.46 ± 7.22 ^c^
Gly	3.32 ± 0.04 ^a^	2.17 ± 0.04 ^c^	2.09 ± 0.13 ^c^	2.92 ± 0.55 ^b^	3.35 ± 1.33 ^a^
Ala	5.11 ± 0.04 ^a^	2.57 ± 0.04 ^c^	2.72 ± 0.16 ^c^	2.15 ± 0.03 ^c^	4.32 ± 0.88 ^b^
Cys	4.64 ± 0.06 ^b^	3.30 ± 0.07 ^c^	4.53 ± 0.27 ^b^	3.74 ± 0.06 ^c^	5.41 ± 0.09 ^a^
Val	6.92 ± 0.08 ^a^	5.50 ± 0.11 ^b^	4.55 ± 0.27 ^c^	4.85 ± 0.08 ^b^	4.15 ± 0.07 ^c^
Met	2.07 ± 0.07 ^a^	2.00 ± 0.04 ^a^	1.51 ± 0.09 ^b^	1.35 ± 0.02 ^c^	1.06 ± 0.02 ^c^
Ile	5.81 ± 0.08 ^a^	3.94 ± 0.08 ^b^	3.28 ± 0.05 ^b^	3.57 ± 0.21 ^b^	2.62 ± 0.04 ^c^
Leu	9.35 ± 0.08 ^a^	5.43 ± 0.11 ^b^	3.49 ± 0.06 ^c^	3.76 ± 0.06 ^c^	1.96 ± 0.03 ^d^
Tyr	4.29 ± 0.08 ^a^	1.94 ± 0.04 ^b^	1.41 ± 0.02 ^b^	1.55 ± 0.09 ^b^	1.50 ± 0.02 ^b^
Phe	4.95 ± 0.07 ^a^	1.65 ± 0.03 ^b^	1.30 ± 0.08 ^c^	1.63 ± 0.10 ^b^	5.35 ± 2.96 ^a^
Lys	8.86 ± 0.20 ^b^	8.53 ± 0.18 ^b^	9.81 ± 0.59 ^a^	9.01 ± 0.54 ^b^	5.15 ± 4.09 ^c^
His	2.89 ± 0.14 ^b^	2.90 ± 0.02 ^b^	3.23 ± 0.19 ^a^	2.94 ± 0.18 ^b^	2.47 ± 0.77 ^b^
Arg	2.08 ± 0.14 ^b^	1.35 ± 0.01 ^b^	1.24 ± 0.07 ^b^	1.48 ± 0.09 ^b^	15.91 ± 10.33 ^a^
Pro	18.13 ± 0.33 ^b^	15.65 ± 0.13 ^c^	18.60 ± 1.11 ^b^	16.75 ± 1.00 ^c^	19.50 ± 0.32 ^a^

^†^ Asp: aspartic acid, Thr: threonine, Ser: serine, Glu: glutamic acid, Gly: glycine, Ala: alanine, Cys: cysteine, Val: valine, Met: methionine, Ile: isoleucine, Leu: leucine, Tyr: tyrosine, Phe: phenylalanine, Lys: lysine, His: histidine, Arg: arginine, Pro: proline. The superscript letters next to each value represent significant groupings. For each amino acid, means with the same letter are not significantly different.

**Table 3:** The effects of isolate NZ 1 and Si on the number of tillers, leaves per tiller, tiller heights, leaf area index (LAI) values, shoot fresh weights, root lengths, and root fresh weights of rice plants. Each value represents a mean ± the standard error of the mean (*n* = 5). Within the same trait, values with the same letter are not significantly different from each other, as evaluated using Tukey’s HSD post hoc tests (*p* < 0.05).

Treatment		Number of Tillers	Leaves Per Tiller	Tiller Height (cm)	Leaf Area Index (LAI)	Shoot Fresh Weight (g)	Root Length (cm)	Root Fresh Weight (g)
Non-stress	Control	6.00 ± 0.70 ^a^	3.54 ± 0.29 ^b^	45.05 ± 0.29 ^a^	8.373 ± 0.38 ^a^	3.89 ± 0.35 ^a^	18.25 ± 1.79 ^b^	4.26 ± 0.37 ^b^
	NZ 1	5.75 ± 0.43 ^a^	3.5 ± 0.24 ^b^	45.50 ± 1.22 ^a^	8.914 ± 1.65 ^a^	3.96 ± 0.28 ^a^	19.50 ± 2.18 ^a^	4.28 ± 0.31 ^b^
	Si	5.5 ± 0.50 ^a^	3.4 ± 0.33 ^b^	44.95 ± 1.51 ^a^	8.920 ± 0.78 ^a^	3.88 ± 0.13 ^a^	18.88 ± 2.46 ^b^	4.72 ± 0.21 ^b^
	NZ 1+Si	6.00 ± 0.00 ^a^	4.00 ± 0.14 ^a^	45.15 ± 0.12 ^a^	8.885 ± 0.03 ^a^	3.98 ± 0.17 ^a^	19.70 ± 2.35 ^a^	4.39 ± 0.25 ^b^
Drought-stressed	Control	4.25 ± 0.43 ^b^	3.45 ± 0.04 ^b^	27.90 ± 4.57 ^c^	4.023 ± 0.26 ^d^	1.78 ± 0.09 ^d^	13.00 ± 1.41 ^d^	2.96 ± 0.21 ^c^
	NZ 1	6.00 ± 0.00 ^a^	3.83 ± 0.14 ^b^	40.05 ± 2.49 ^b^	6.241 ± 0.04 ^c^	2.95 ± 0.33 ^c^	16.75 ± 1.09 ^c^	5.18 ± 0.33 ^a^
	Si	5.5 ± 0.5 ^a^	3.67 ± 0.27 ^b^	39.40 ± 1.06 ^b^	6.392 ± 0.13 ^c^	2.88 ± 0.19 ^c^	17.23 ± 0.77 ^b^	5.31 ± 0.27 ^a^
	NZ 1+Si	6.00 ± 0.00 ^a^	3.50 ± 0.00 ^b^	43.15 ± 1.84 ^b^	7.069 ± 0.75 ^b^	3.31 ± 0.21 ^b^	18.75 ± 2.49 ^b^	5.98 ± 0.31 ^a^

## Data Availability

Data will be made available on request.

## Supplementary Materials

The following supporting information can be downloaded at https://www.mdpi.com/article/10.3390/microorganisms13051046/s1, Figure S1: Visual representation of (A) siderophores production, (B) catalase production, (C) exopolysaccharide production, and (D) phosphate solubilizing activity exhibited by the isolates; Table S1: Gene names and their corresponding forward with reverse primers; Figure S2: Visual representation of (A) siderophores, (B) catalase, (C) exopolysaccharide productions and (D) phosphate solubilization activities of the five isolates, which were later renamed as written in the box; Figure S3: Performances of the isolates (A) NZ 1, (B) NZ 2, (C) NZ 3, (D) MIJ 1 and (E) MIJ 2 on different concentrations of PEG-6000 levels in 4 continuous intervals of 8 hours; Figure S4: Phylogenetic tree of *Pseudomonas qingdaonensis* NZ 1 with closely related species, inferred using the Neighbour-Joining method. 16s rRNA of *P. qingdaonensis* NZ 1 was compared with sequences with high similarity values acquired from NCBI blast program The percentage of replicate trees in which the associated taxa clustered together in the bootstrap test (1000 replicates) are shown next to the branches. The tree is drawn to scale, with branch lengths in the same units as those of the evolutionary distances used to infer the phylogenetic tree. The evolutionary distances were computed using the p-distance method and are in the units of the number of base differences per site. There were a total of 1490 positions in the final dataset. Evolutionary analyses were conducted in MEGA11. Supplemental material encompasses gene primer list, plant growth-promoting traits screening of the isolates, temporal changes in optical density of the isolates grown in PEG-6000, and phylogenetic tree of isolate *Pseudomonas qingdaonensis* NZ 1 with closely related species.

## Abbreviation

ABA	Abscisic Acid
JA	Jasmonic Acid
PGPR	Plant Growth-Promoting Rhizobacteria
ROS	Reactive Oxygen Species
SA	Salicylic Acid
