# Supplementary material for: New Insights into the Synergistic Interaction Between Pseudomonas qingdaonensis NZ 1 and Silicon to Mitigate Drought Stress in Rice
Source: Microorganisms. 2025 Apr 30;13(5):1046. doi: 10.3390/microorganisms13051046 (PMC12114193; doi:10.3390/microorganisms13051046)
Supplement: Supplementary file 1 [file microorganisms-13-01046-s001.zip › microorganisms-3521237-supplementary.pdf]

Supplementary Material

Supplemental Table S1: Gene names and their corresponding forward with reverse primers.

Gene name	Forward primer	Reverse primer
OsNCED3	5`CTCACATACAGCGGCAGCAC`3	5`CGCTCGAGGACATTCGCCAC`3
OsCYP707A6	5`AGACGAGGAGCATGACACT`3	5`CGGGTTGTGATGGATGTT`3
OsbZIP23	5`CTGTCGTCGCTGTCGCCATC`3	5`GATCATCCGCCGCTGTCTTCTC `3
OsDREB1B	5`ACAGAGTAGGCAATGAGACTG AGGAT`3	5`TTACAGGAATTCATTGACTGC ACAT`3
OsLSi1	5`ACGAGATGTCGTCGATCGTG`3	5`ACGAGATGTCGTCGATCGTG`3

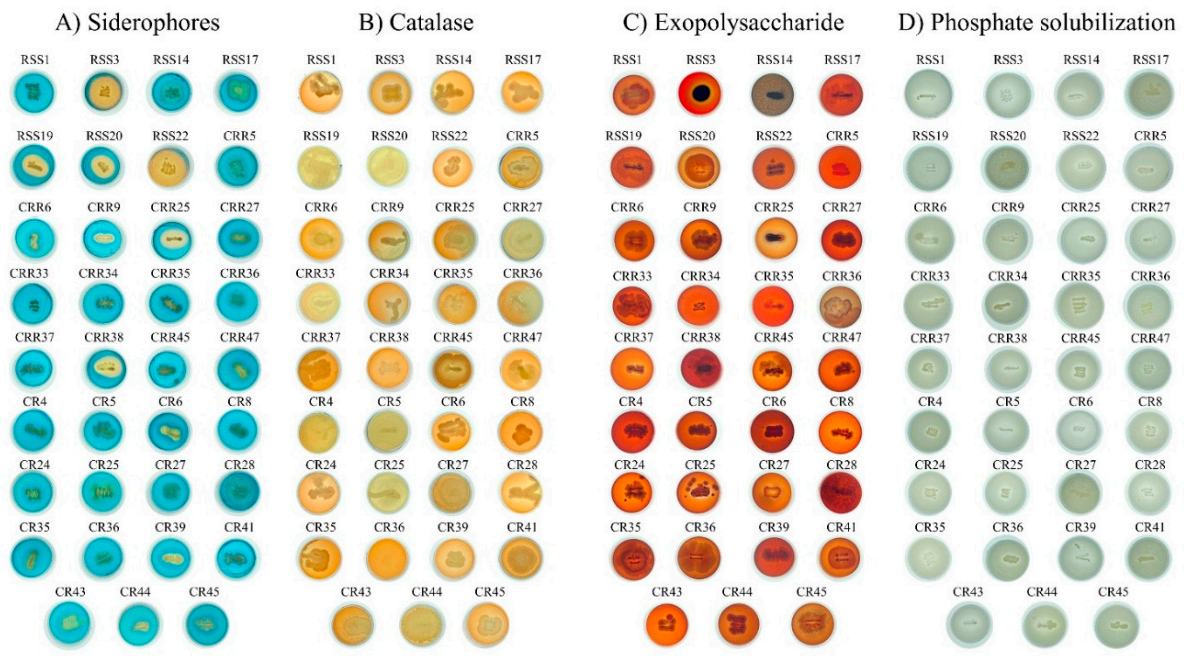

Supplemental Figure S1 Visual representation of A) siderophores production, B) catalase production, C) exopolysaccharide production, and D) phosphate solubilizing activity exhibited by the isolates.

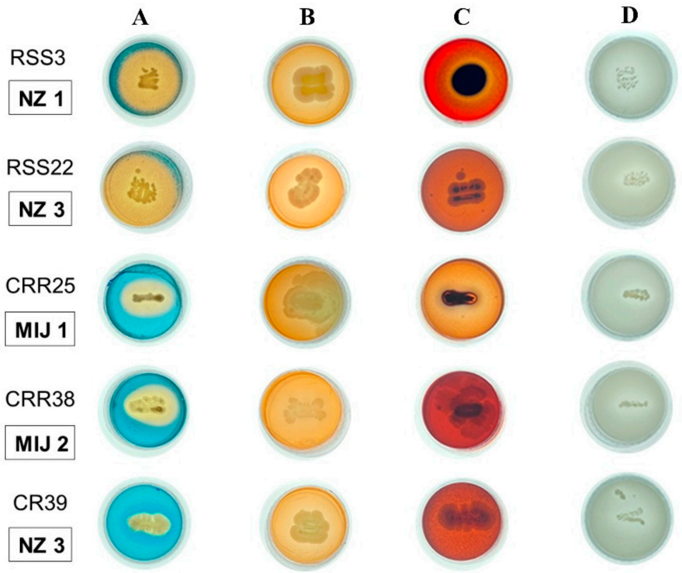

Supplemental Figure S2 Visual representation of A) siderophores, B) catalase, C) exopolysaccharide productions and D) phosphate solubilization activities of the five isolates, which were later renamed as written in the box.

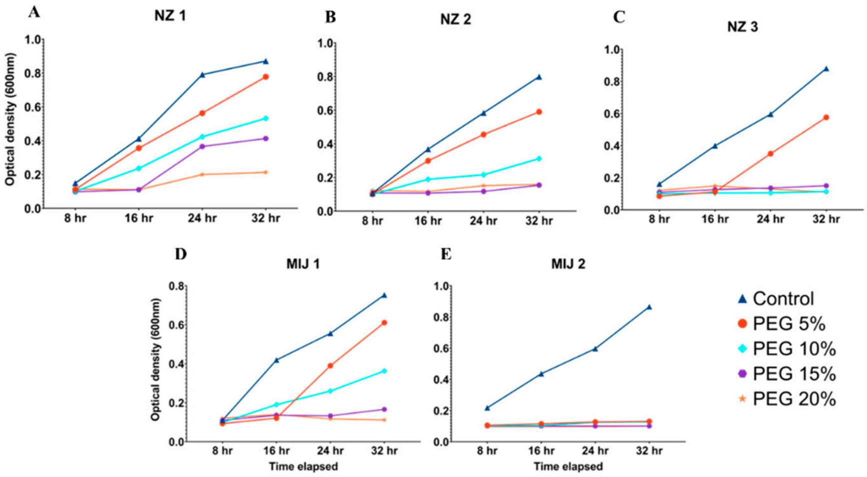

Supplemental Figure S3 Performances of the isolates A) NZ 1, B) NZ 2, C) NZ 3, D) MIJ 1 and E) MIJ 2 on different concentrations of PEG-6000 levels in 4 continuous intervals of 8 hours.

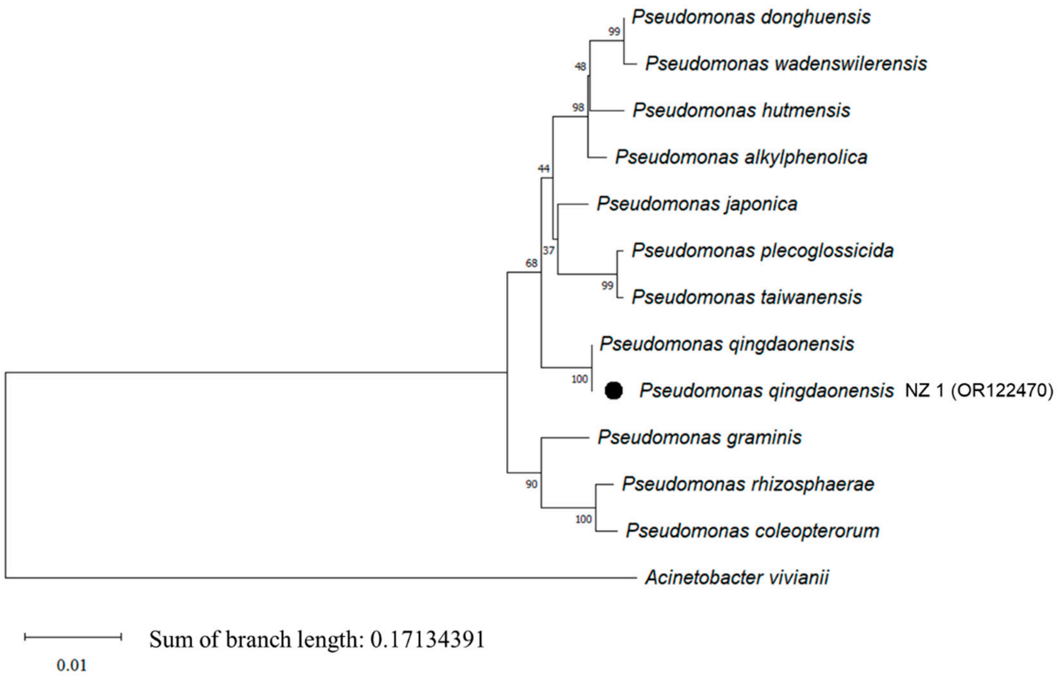

Supplemental Figure S4 Phylogenetic tree of Pseudomonas qingdaonensis NZ 1 with closely related species, inferred using the Neighbour-Joining method. 16s rRNA of P. qingdaonensis NZ 1 was compared with sequences with high similarity values acquired from NCBI blast program The percentage of replicate trees in which the associated taxa clustered together in the bootstrap test (1000 replicates) are shown next to the branches. The tree is drawn to scale, with branch lengths in the same units as those of the evolutionary distances used to infer the phylogenetic tree. The evolutionary distances were computed using the p-distance method and are in the units of the number of base differences per site. There were a total of 1490 positions in the final dataset. Evolutionary analyses were conducted in MEGA11.
